# Hyper-stable organo-Eu^III^ luminophore under high temperature for photo-industrial application

**DOI:** 10.1038/srep24458

**Published:** 2016-04-14

**Authors:** Ayako Nakajima, Takayuki Nakanishi, Yuichi Kitagawa, Tomohiro Seki, Hajime Ito, Koji Fushimi, Yasuchika Hasegawa

**Affiliations:** 1 Faculty of Engineering, Hokkaido University, N13 W8, Kita-ku, Sapporo, Hokkaido 060-8626, Japan

## Abstract

Novel organo-Eu^III^ luminophores, Eu(hfa)_x_(CPO)_y_ and Eu(hfa)_x_(TCPO)_y_ (hfa: hexafluoroacetylacetonate, CPO: 4-carboxyphenyl diphenyl phosphine oxide, TCPO: 4,4′,4″-tricarboxyphenyl phosphine oxide), were synthesized by the complexation of Eu^III^ ions with hfa moieties and CPO or TCPO ligands. The thermal and luminescent stabilities of the luminophores are extremely high. The decomposition temperature of Eu(hfa)_x_(CPO)_y_ and Eu(hfa)_x_(TCPO)_y_ were determined as 200 and 450 °C, respectively. The luminescence of Eu(hfa)_x_(TCPO)_y_ under UV light irradiation was observed even at a high temperature, 400 °C. The luminescent properties of Eu(hfa)_x_(CPO)_y_ and Eu(hfa)_x_(TCPO)_y_ were estimated from emission spectra, quantum yields and lifetime measurements. The energy transfer efficiency from hfa moieties to Eu^III^ ions in Eu(hfa)_x_(TCPO)_y_ was 59%. The photosensitized luminescence of hyper-stable Eu(hfa)_x_(TCPO)_y_ at 400 °C is demonstrated for future photonic applications.

There has been significant interest in the development of luminescent lanthanide materials for use in devices such as fluorescent lamps^1^,^2^,^3^, LED lights^4^,^5^,^6^,^7^,^8^,^9^,^10^,^11^ and displays^10^,^11^,^12^,^13^. Recently, we have focused on organo lanthanide luminophores with strong luminescent properties for a future energy saving measures^14^. The organo lanthanide luminophores are attached with aromatic antenna for high photon absorption efficiency. The general organic luminophores are decomposed under 200 °C unfortunately. In the case of industrial applications of organic devices using luminescent lanthanide materials, thermostability is required for effective material production process and long term durability. This manuscript describes new organo lanthanide luminophores with thermostability and strong luminescent properties using a photosensitized effect. The organo lanthanide luminophore at 400 °C is inconceivable material, which is put on a characteristics of solid ceramics and smart molecules.

There are currently various types of organo lanthanide luminophores based on characteristic ligand design that have been developed as strongly luminescent materials^14^,^15^,^16^,^17^,^18^,^19^,^20^,^21^,^22^,^23^,^24^,^25^,^26^,^27^,^28^,^29^,^30^,^31^,^32^,^33^,^34^,^35^. A three-dimensional networks composed of organo lanthanide luminophores, which prevent stretching vibration and rotations of organic ligands, leads to a thermostable structure. Du and coworkers have synthesized a three-dimensional lanthanide compound with 1,3-benzenedicarboxylic acid for the construction of a thermostable structure^36^. Hong and coworkers have demonstrated that a three-dimensional lanthanide metal-organic framework (MOF) composed of lanthanide ions (Ln^III^ = Nd, Sm, Eu, Gd) and tris-(4-carboxylphenyl)phosphine oxide has a high decomposition temperature (500 °C)^37^. However, the benzene-typed joint ligands do not promote effective photosensitization in organo-Eu^III^ luminophores (*η* < 1%). Thermostable lanthanide luminophores with effective photosensitization are expected to open up a new field of luminescent material science. We have attempted to prepare an organo lanthanide material with high thermostability and effective photosensitized luminescence. In this study, novel organo-Eu^III^ luminophores with hfa moieties (hfa: hexafluoroacethylacetonato) and carboxy phosphine oxide (CPO: 4-carboxyphenyl diphenyl phosphine oxide/TCPO: 4,4′,4″-tricarboxyphenyl phosphine oxide) are reported, the structures of which are shown in Fig. 1. The hfa moieties act as photosensitization ligands in organo-Eu^III^ luminophores and play an important role in the suppression of non-radiative transition via vibrational relaxation due to their lower vibrational frequencies^38^. Coordination of the phosphine oxide parts in CPO and TCPO as three-dimensional joint ligands provides a low-vibrational frequency for strong luminescence. The CPO and TCPO ligands are also designed to include carboxy groups for construction of the thermostable Ln-MOF structure reported by Hong and coworkers^37^. A mononuclear Eu^III^ complex, Eu(hfa)_3_(TPPO)_2_ (TPPO: triphenylphosohine oxide) was prepared as a standard reference. The thermostability of the organo-Eu^III^ luminophores was evaluated using thermogravimetric analysis (TGA). The luminescent properties were estimated from emission spectra, quantum yields and lifetime measurements. The bright luminescence of Eu(hfa)_x_(TCPO)_y_ at 400 °C was successfully observed and the energy transfer efficiency of Eu(hfa)_x_(TCPO)_y_ was calculated to be 47%. Thus, thermostable and effective photosensitized organo-Eu^III^ luminophores were demonstrated for the first time.

## Results and Discussion

### Thermostable Properties

**Figure 2:**
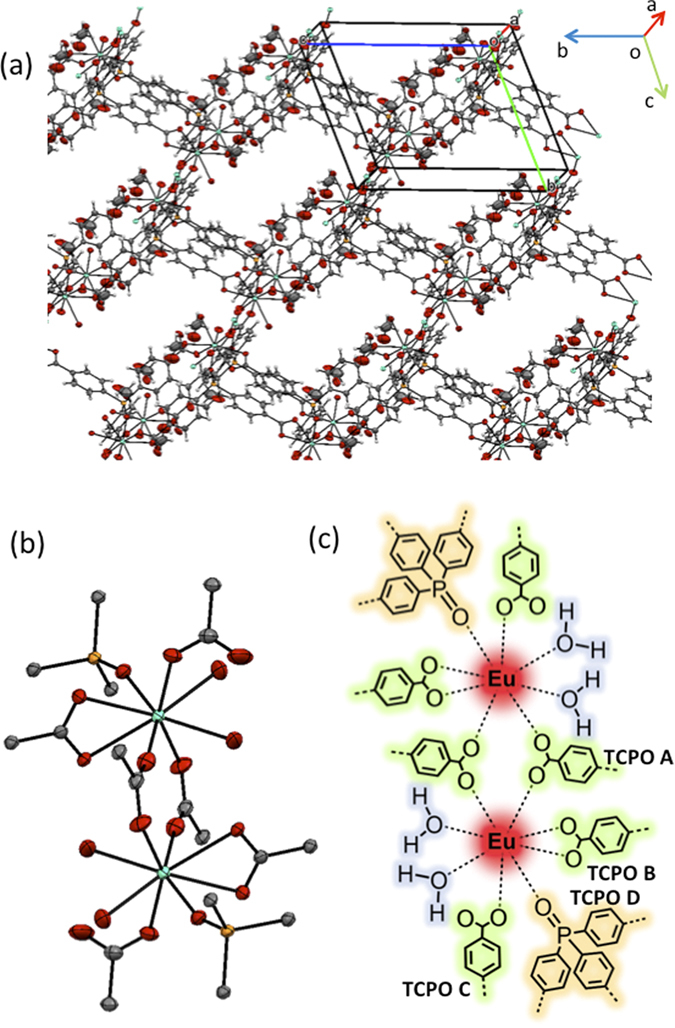
(**a,b**) ORTEP views of Eu(hfa)_x_(TCPO)_y_ consisted of Eu^III^ ions and TCPO ligands, (**c**) chemical structure of Eu^III^ coordination sites.

**Figure 3:**
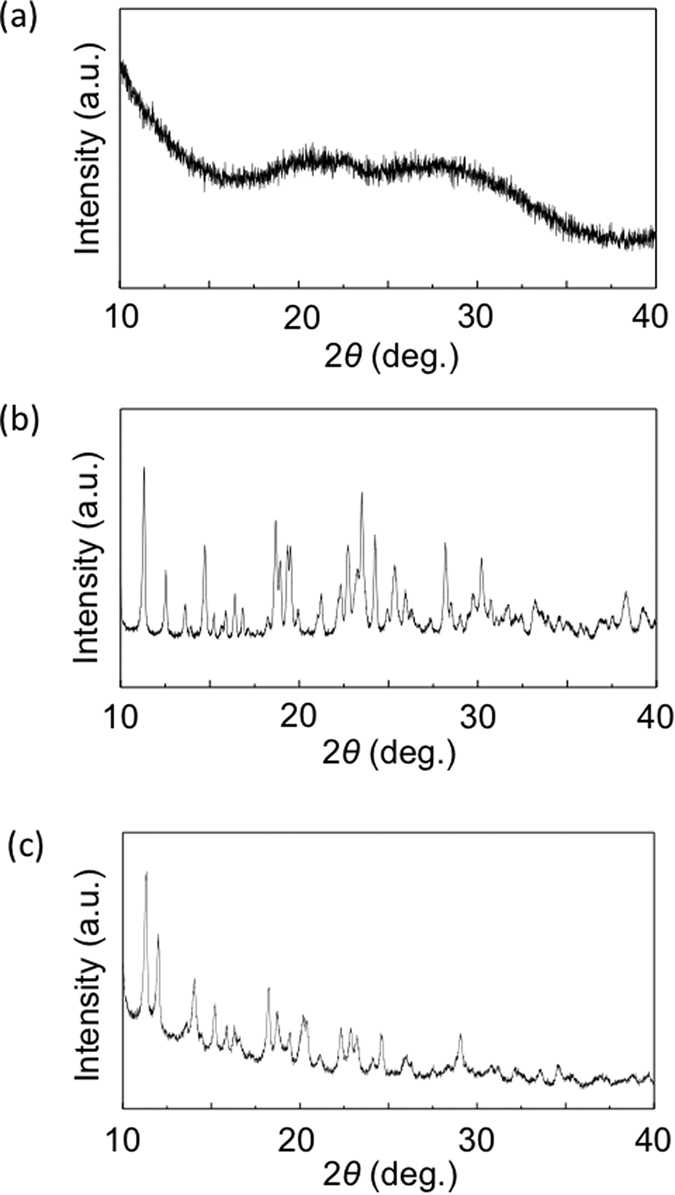
XRD patterns of (**a**) Eu(hfa)_x_(CPO)_y_, (**b**) as-prepared Eu(hfa)_x_(TCPO)_y_ and (**c**) Eu(hfa)_x_(TCPO)_y_ under heat treatment at 90 °C.

In previous work, Eu^III^ luminophore with carboxy phosphine oxide have been reported^37^. The material has no photosensitized hfa moiety. Eu(hfa)_x_(TCPO)_y_ and Eu(hfa)_x_(CPO)_y_ were synthesized by the complexation of the carboxy phosphine oxide (CPO or TCPO) with Eu(hfa)_3_(H_2_O)_2_ in methanol under reflux. The phosphine oxide parts (P = O) and the carboxy groups (COO^−^) in CPO and TCPO ligands effectively promote the formation of polymeric structures. The significant vibrational bands at C=O and P=O groups of Eu(hfa)_x_(CPO)_y_ were shifted to shorten wavenumbers (1658 and 1143 cm^−1^) (CPO ligand: 1702 and 1151 cm^−1^). The IR bands of Eu(hfa)_x_(TCPO)_y_ were also observed at 1622 and 1102 cm^−1^, which are shorter than those of the ligand (TCPO ligand: 1692 and 1115 cm^−1^) (see Supplementary Information, Fig. S1). We successfully synthesized Eu(hfa)_x_(TCPO)_y_ without base condition. This chelate reaction is a new method for preparation of Eu(hfa)_x_(TCPO)_y_. On the other hand, Eu(hfa)_x_(CPO)_y_ is prepared under base-condition (addition of triethyl amine). The reaction difference is might be due to moiety of the joint ligands, CPO and TCPO. The x and y in formulas in Eu(hfa)_x_(CPO)_y_ and Eu(hfa)_x_(TCPO)_y_ are defined 0 < x < 1 and 0 < y < 3. We estimated x = 0.38, y = 2.12 in Eu(hfa)_x_(CPO)_y_ and x = 0.03, y = 1.92 in Eu(hfa)_x_(TCPO)_y_ using EDX data (see Supplementary Information, Fig. S2). In order to identify the structure of Eu(hfa)_x_(TCPO)_y_, we tried to measure by single-crystal X-ray structure analysis. The structure was determined to be eight-coordinated structure with two water molecules and five TCPO ligands. The two TCPO ligands show bidentate bridged connection between two Eu^III^ ions (TCPO A in Fig. 2). We also found that two TCPO ligands show bidentate (TCPO B) and monodentate (TCPO C) connection in one Eu^III^ ion. Final TCPO ligand is attached to one Eu^III^ ion by P=O group (TCPO D). The Eu(hfa)_x_(TCPO)_y_ crystal provides three dimensional network structure. This single crystal is including four methanol molecules in one unit (Fig. 2 and Table 1). These structures of the polymeric compounds were analyzed using X-ray diffraction (XRD) measurements. Figure 3 shows XRD patterns for both luminophores. Broad peaks were observed for Eu(hfa)_x_(CPO)_y_ at around 20° and 28° (Fig. 3a). The Eu(hfa)_x_(CPO)_y_ has an amorphous structure at room temperature. In contrast, the as-prepared white powder of Eu(hfa)_x_(TCPO)_y_ has noticeable peaks at 11.29°, 12.41°, 13.45°, 14.76°, 18.88°, 19.47°, 22.62°, 23.44°, 24.23°, 25.41°, 28.48°, and 30.11° (Fig. 3b), and Eu(hfa)_x_(TCPO)_y_ after heat treatment (90 °C, 2 h, under reduced pressure) also has noticeable peak at 11.32°, 11.99°, 14.05°, 15.21°, 18.22°, 18.73°, 20.21°, 20.37°, 22.29°, 22.88°, 23.23°, and 29.07° (Fig. 3c). Thus, it is considered that the triphenylphosphine oxide with three carboxy groups, the TCPO joint ligand, leads to the formation of a crystalline structure, and the structure change by heat treatment. We have checked the XRD of Eu(hfa)_x_(TCPO)_y_ compared with that of Eu((CH_3_)_2_NCHO)_x_(TCPO)_y_ in previous work^37^ (see Supplementary Information, Fig. S3). The XRD patterns of Eu(hfa)_x_(TCPO)_y_ is much different from that of Eu((CH_3_)_2_NCHO)_x_(TCPO)_y_. Identification of the polymeric structure was performed using fast atom bombardment-mass spectrometry (FAB-MS) and energy dispersive X-ray spectroscopy (EDX) measurements. The fragment peaks of Eu(hfa)_x_(CPO)_y_ and Eu(hfa)_x_(TCPO)_y_ in the FAB-MS spectra agree with those calculated for [Eu_2_(hfa)_3_(CPO)_2_]^+^ and [Eu(hfa)_2_(TCPO)·5H_2_O]^+^ fragments, respectively (see Supplementary Information Fig. S4). According to the determination of element ratio, we estimated the Eu(Mα), P(Kα) and F(Kα) of Eu(hfa)_x_(CPO)_y_ and Eu(hfa)_x_(TCPO)_y_ for EDX measurements calibrated with Eu(hfa)_3_(TPPO)_2_ as a standard. The EDX measurements indicated the percentage of hfa moieties in Eu(hfa)_x_(CPO)_y_ and Eu(hfa)_x_(TCPO)_y_ were 10.8% and 0.89%, respectively. We propose that the small amount of hfa molecules attached on the crystal surface. The hfa molecules on the surface were successfully detected by ionized-fragment information using FAB-MS spectrum (Fig. S4b Eu(hfa)_2_TCPO·5H_2_O). In contrast, the EDX signals of the XRF measurement gave the average information about total element ratio of Eu(hfa)_x_(TCPO)_y_.

**Figure 4:**
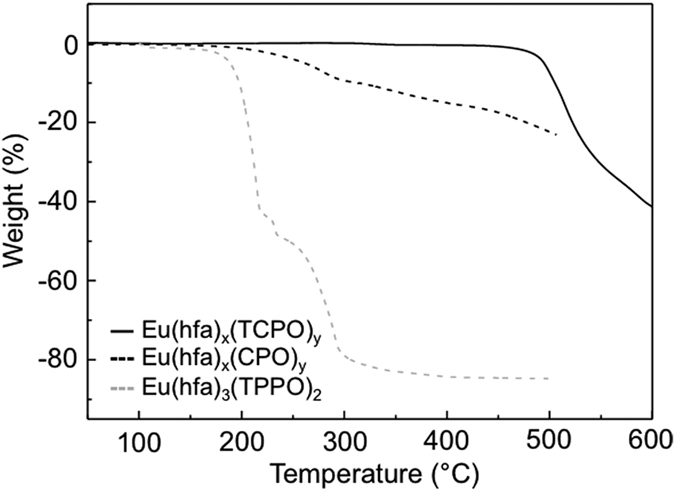
TGA profiles of Eu(hfa)_x_(TCPO)_y_ (black line), Eu(hfa)_x_(CPO)_y_ (black dot line), and Eu(hfa)_3_(TPPO)_2_ (gray dot line) under an argon atmosphere.

The thermo-stabilities of the Eu(hfa)_x_(CPO)_y_ and Eu(hfa)_x_(TCPO)_y_ polymeric structures were evaluated using TGA and the results are shown in Fig. 4. The TGA profile for the luminescent mononuclear Eu^III^ complex, Eu(hfa)_3_(TPPO)_2_, was also measured as a standard reference. The decomposition temperature of Eu(hfa)_3_(TPPO)_2_ was 200 °C. The weight of Eu(hfa)_x_(CPO)_y_ gradually decreases from 200 °C, which may be due to the loose packing structure in amorphous Eu(hfa)_x_(CPO)_y_ to promote partial elimination of the hfa moieties. The decomposition temperature of Eu(hfa)_x_(TCPO)_y_ was 450 °C. We cannot observed the elimination of solvent from the material. This result indicates that Eu(hfa)_x_(TCPO)_y_ have no solvent in the structure after heat treatment. Therefore, XRD measurements of Eu(hfa)_x_(TCPO)_y_ were kept under 450 °C (see Supplementary Information, Fig. S5). The decomposition temperature of Eu(hfa)_x_(TCPO)_y_ is the highest among the organo-Eu^III^ luminophores with photosensitized hfa moieties. Thus, a luminescent organo-Eu^III^ luminophore with extra-high thermostability was successfully synthesized.

**Figure 5:**
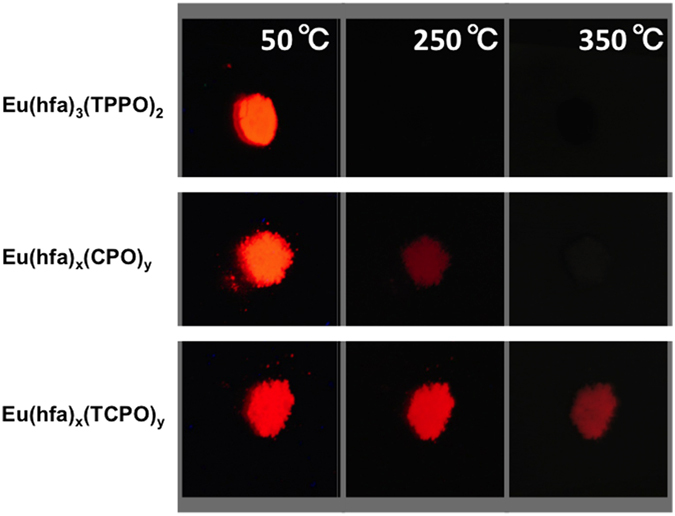
Photographs of Eu(hfa)_3_(TPPO)_2_, Eu(hfa)_x_(CPO)_y_ and Eu(hfa)_x_(TCPO)_y_ at 50 °C, 250 °C, and 350 °C heating on the hot plate under UV light irradiation (*λ* = 365 nm).

The luminescence images for Eu(hfa)_3_(TPPO)_2_, Eu(hfa)_x_(CPO)_y_, and Eu(hfa)_x_(TCPO)_y_ heated on a hot plate under UV light irradiation (λ = 365 nm) are shown in Fig. 5. Eu(hfa)_3_(TPPO)_2_ exhibits red luminescence at 50 °C but does not emit photons at 250 °C due to their thermal decomposition. The red luminescence of Eu(hfa)_x_(CPO)_y_ faded out at around 250 °C. In contrast, bright red luminescence was successfully observed from Eu(hfa)_x_(TCPO)_y_ under 400 °C. (See emission spectra under control of temperature in Supplementary Information, Fig. S6a). Thus, Eu(hfa)_x_(TCPO)_y_ exhibits both effective photosensitized luminescence and thermostability.

### Luminescent Properties

**Figure 6:**
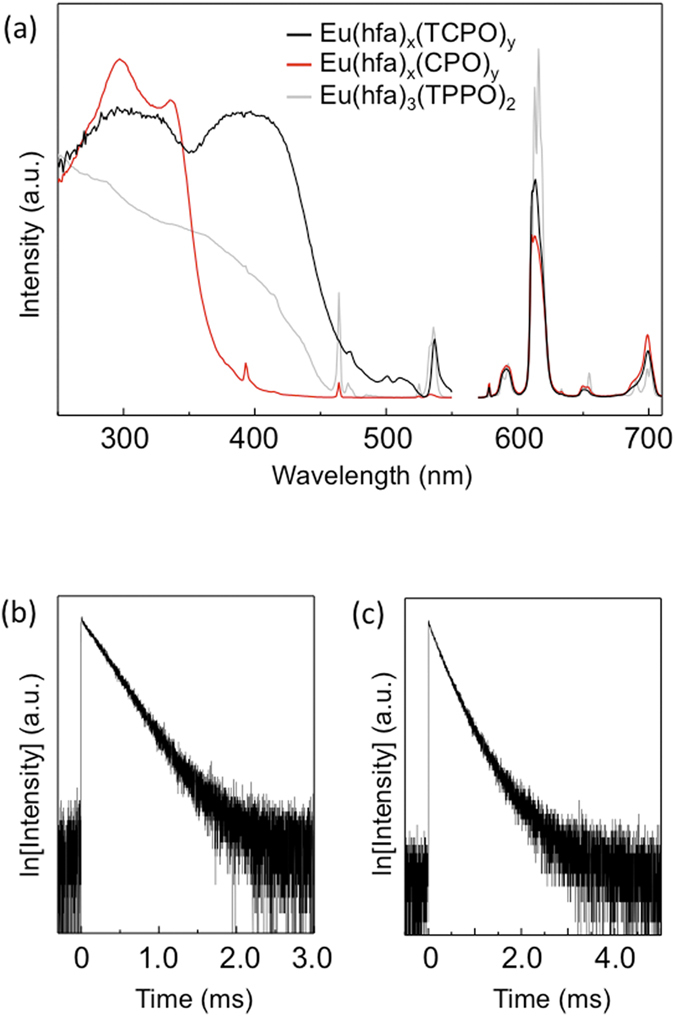
(**a**) Excitation and Emission spectra of Eu(hfa)_x_(TCPO)_y_ (black line), Eu(hfa)_x_(CPO)_y_ (red line) and Eu(hfa)_3_(TPPO)_2_ (gray line) excited at 365 nm in the solid state, Decay profile of (**b**) Eu(hfa)_x_(CPO)_y_, and (**c**) Eu(hfa)_x_(TCPO)_y_ in the solid state.

Excitation and emission spectra for Eu(hfa)_3_(TPPO)_2_, Eu(hfa)_x_(CPO)_y_ and Eu(hfa)_x_(TCPO)_y_ in the solid state detected at 613.5 nm and excited at 365 nm are shown in Fig. 6. The excitation bands of Eu(hfa)_x_(CPO)_y_ at around 300 nm is assigned to π–π^*^ transition of hfa moieties^39^. We also found characteristic excitation band at around 400 nm in Eu(hfa)_x_(TCPO)_y_ crystals. The emission bands were observed at around 578, 591, 613, 651, and 699 nm, which are attributed to the 4*f*-4*f* transitions of Eu^III^ (^5^D_0_–^7^F_J_: *J* = 0, 1, 2, 3, and 4, respectively). The spectra were normalized with respect to the magnetic dipole transition intensities at 591 nm (Eu^III^: ^5^D_0_—^7^F_1_), which is known to be insensitive to the surrounding environment of the lanthanide ions. The normalized emission intensity of Eu(hfa)_x_(TCPO)_y_ at 613 nm is larger than that of Eu(hfa)_x_(CPO)_y_. These spectral shapes of Eu(hfa)_x_(TCPO)_y_ and Eu(hfa)_x_(CPO)_y_ are different from that of crystalline Eu(hfa)_3_(TPPO)_2_.

Time-resolved emission profiles of Eu(hfa)_x_(CPO)_y_ and Eu(hfa)_x_(TCPO)_y_ are shown in Fig. 6b,c, respectively. The emissions from Eu(hfa)_x_(CPO)_y_ indicates single-exponential decays of millisecond scale. We estimated the emission lifetime of Eu(hfa)_x_(TCPO)_y_ using single-exponential decay. The lifetime and R^2^ under single-exponential analysis were found to be 0.61 ms and 0.996, respectively. The lifetime is similar to that of Eu(hfa)_x_(CPO)_y_. In this paper, we used the single-exponential analysis for estimation of lifetime of Eu(hfa)_x_(TCPO)_y_. We consider that the luminescence of Eu(hfa)_x_(TCPO)_y_ comes from one-dominant Eu^III^ species with hfa moieties on the crystal surface. The emission lifetimes of Eu(hfa)_3_(TPPO)_2_, Eu(hfa)_x_(CPO)_y_ and Eu(hfa)_x_(TCPO)_y_ were determined to be 0.80, 0.60 and 0.61 ms, respectively.

The 4*f *− 4*f* emission quantum yields (Φ_4*f*−4*f*_) and the radiative (*k*_r_) and non-radiative (*k*_nr_) rate constants of these Eu^III^ compounds were calculated using the following equations.




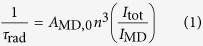







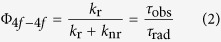







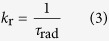







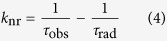




The radiative lifetime (*τ*_rad_) is defined as an ideal emission lifetime without non-radiative processes. The radiative lifetime is expressed by equation 1, where *A*_MD,0_ is the spontaneous emission probability for the ^5^D_0_—^7^F_1_ transition *in vacuo* (14.65 s^−1^), *n* is the refractive index of the medium (an average index of refraction equal to 1.5 was employed), and (*I*_tot_/*I*_MD_) is the ratio of the total area of the corrected Eu^III^ emission spectrum to the area of the ^5^D_0_—^7^F_1_ band. The emission quantum yields, and the radiative and non-radiative rate constants are summarized in Table 2.

The emission quantum yield of Eu(hfa)_x_(TCPO)_y_ excited at 355 nm (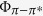: *π* − π^*^ transition band of hfa moieties) was also measured to calculate the energy transfer efficiency (*η*), which was determined as 34%. The energy transfer efficiency of Eu(hfa)_x_(TCPO)_y_ (decomposition temperature = 450 °C, *η* = 59%) is larger than that of the previously reported thermostable organo-Eu^III^ luminophore, [Eu(hfa)_3_(dpbp)]_n_ (dpbp: 4,4′-bis(diphenyl phospholyl)biphnyl, decomposition temperature = 308 °C, *η* = 40%)^40^.

## Summary and Conclusions

A organo-Eu^III^ luminophore, Eu(hfa)_x_(TCPO)_y_, with effective thermostability and photosensitized luminescent property was successfully synthesized. Thermostable Eu(hfa)_x_(TCPO)_y_ exhibits bright red luminescence at 400 °C under UV light irradiation. The luminescence of Eu(hfa)_x_(TCPO)_y_ is due to photosensitized energy transfer from hfa moieties to Eu^III^ ions. Thermostable organo-lanthanide luminophores are expected to open up the frontier fields of photophysical science, material chemistry and industrial applications.

## Experimental Section

### Materials

Europium acetate *n*-hydrate (99.9%), diphenyl(*p*-tolyl)phosphine and tri-*p*-tolylphosphine were purchased from Wako Pure Chemical Industries Ltd. Hexafluoroacetylacetone and triphenylphosphine oxide (TPPO) were obtained from Tokyo Kasei Organic Chemicals Co., Inc. Dimethyl sulfoxide-*d*_*6*_ (D, 99.9%) was obtained from Kanto Chemical Co., Inc. All other chemicals and solvents were reagent grade and were used without further purification.

### Apparatus

^1^H NMR (400 MHz) spectra were recorded on a JEOL ECS400. Chemical shifts were reported in δ ppm, referenced to an internal tetramethylsilane standard for ^1^H NMR spectroscopy. Infrared spectra were measured using a Thermo Nicolet AVATAR 320 FT-IR spectrometer. FAB-MS spectra were recorded on a JEOL JMS-700TZ. Elemental analyses were performed on a J-Science Lab Micro Corder JM 10 and an Exeter Analytical CE440. In addition, the ratio of F to Eu was measured using Energy Dispersive X-ray Fluorescence Spectrometer EDX-8000 with (reference material: Eu(hfa)_3_(TPPO)_2_). XRD patterns were characterized by a RIGAKU SmartLab X-ray diffractometer with Cu Kα radiation, a D/teX Ultra detector, and a temperature control unit (Anton Paar, TCU-110). Thermogravimetric Analysis was performed on a Seiko Instruments Inc. EXSTAR 6000 (TG-DTA 6300) at first heating rate of 10 °C min^−1^ up to 100 °C, cooling rate of 10 °C min^−1^ up to 40 °C, and second heating rate of 1 °C min^−1^ up to 500 °C.

### Preparation of 4-carboxyphenyl diphenyl phosphine oxide (CPO)

CPO was synthesized by the oxidation of diphenyl(*p*-tolyl)phosphine with potassium permanganate, according to the procedure described in the literature^41^. Yield: 54%; ^1^H NMR (400 MHz, DMSO-*d*_*6*_, 298K): δ 8.10—8.06 (*dd*, 2H), 7.78—7.72 (*dd*, 2H), 7.67—7.61 (*m*, 6H), 7.60—7.54 (*td*, 4H) ppm; IR (ATR): 1658, 1592, 1540, 1498, 1411, 1254, 1144, 1118 cm^−1^; Elemental analysis calcd (%) for C_19_H_15_O_3_P: C 70.81, H 4.69; found: C 70.15, H 4.49.

### Preparation of 4,4′,4″-tricarboxyphenyl phosphine oxide (TCPO)

TCPO was synthesized by the oxidation of tri-*p*-tolylphosphine with potassium permanganate, according to the procedure described in the literature^42^. Yield: 34%; ^1^H NMR (400 MHz, DMSO-*d*_*6*_, 298K): δ 8.12—8.08 (*dd*, 6H), 7.88—7.75 (*dd*, 6H) ppm; IR (ATR): 1692, 1395, 1246, 1162, 1102, 1016 cm^−1^; Elemental analysis calcd (%) for [C_21_H_15_O_7_P + H_2_O]: C 58.89, H 4.00; found: C 58.67, H 4.08.

### Preparation of [Eu(hfa)_3_(H_2_O)_2_]

Europium acetate *n*-hydrate (5.0 g, 12 mmol) was dissolved in distilled water (20 mL). A solution of hexafluoroacetylacetone was added dropwise to the solution. The reaction mixture produced a precipitation of white yellow powder after stirring for 3 h at room temperature. The reaction mixture was filtered, and the resulting powder was used without further purification for next step. Yield: 95%; IR (KBr): 1650, 1258–1145 cm^−1^; Elemental analysis calcd (%) for C_15_H_7_EuF_18_O_8_: C 22.27, H 0.87; found: C 22.12, H 1.01^42^,^43^.

### Preparation of Eu(hfa)_3_(TPPO)_2_

Methanol (100 mL) containing Eu(hfa)_3_(H_2_O)_2_ (4.28 g, 6 mmol) and TPPO (2.78 g, 10 mmol) was refluxed under stirring for 12 h. The reaction mixture was concentrated using a rotary evaporator. Reprecipitation by addition of excess hexane solution produced crude crystal, which were washed in toluene several times. Recrystallization from hot toluene/cyclohexane gave white needle crystals. Yield: 74%; ^1^H-NMR (400 MHz, CD_3_-COCD_3_, TMS): δ = 5.42 (*s*, 3H), 7.58—7.71 (*m*, 12H), 7.76—7.86 (*m*, 6H), 8.67 (*br*, 12H) ppm; IR (KBr): 1650, 1250—1150, 1125 cm^−1^; Elemental analysis calcd (%) for C_51_H_33_EuF_18_O_8_P_2_: C 46.07, H 2.50; found: C 45.94, H 2.57^38^.

### Preparation of Eu(hfa)_x_(CPO)_y_

CPO (207 mg, 0.64 mmol) and Eu(hfa)_3_(H_2_O)_2_ (720 mg, 0.89 mmol) were dispersed in methanol (30 mL), and triethylamine was added to neutralize. The dispersion was stirred for 5 h at 60 °C. The precipitate was washed with methanol several times, and dried *in vacuo*. Yield: 45.3 mg; IR (ATR) 1658, 1592, 1540, 1498, 1411, 1254, 1144, 1118 cm^−1^; FAB-MS (m/z): [Eu_2_(hfa)_3_(CPO)_2_]^+^ calcd for C_53_H_31_Eu_2_F_18_O_12_P_2_, 1566.9; found 1566.7; EDX found (%): CPO, 60.0; Eu, 28.3; hfa, 10.7.

### Preparation of Eu(hfa)_x_(TCPO)_y_

TCPO (260 mg, 0.63 mmol) and Eu(hfa)_3_(H_2_O)_2_ (720 mg, 0.89 mmol) were dispersed in methanol (30 mL). The dispersion was stirred for 9 h at 60 °C. The white precipitate was washed with methanol several times, and dried *in vacuo* oven at 90 °C (see Supplementary Information, Fig. S8). Yield: 294.7 mg; IR (ATR) 1624, 1548, 1398, 1382, 1185, 1145, 1116, 1050, 1018 cm^−1^; FAB-Mass (m/z): [Eu(hfa)_2_(TCPO)·5H_2_O]^+^ calcd for C_31_H_27_EuF_12_O_16_P, 1067.01; found 1067.3; EDX found (%): TCPO, 64.9; Eu, 33.8; hfa, 0.9.

### Optical measurements

Emission spectra were recorded on a HORIBA Fluorolog-3 spectrofluorometer and corrected for the response of the detector system. Emission lifetimes (*τ*_obs_) were measured using the third harmonics (355 nm) of a Q-switched Nd:YAG laser (Spectra Physics, INDI-50, fwhm = 5 ns, *λ* = 1064 nm) and a photomultiplier (Hamamatsu photonics, R5108, response time ≤1.1 ns). The Nd:YAG laser response was monitored with a digital oscilloscope (Sony Tektronix, TDS3052, 500 MHz) synchronized to the single-pulse excitation. The emission quantum yield excited at 355 nm (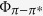) was estimated using a JASCO F-6300-H spectrometer attached with JASCO ILF-53 integrating sphere unit (φ = 100 mm).

## Additional Information

**How to cite this article**: Nakajima, A. *et al.* Hyper-stable organo-EuIII luminophore under high temperature for photo-industrial application. *Sci. Rep.* **6**, 24458; doi: 10.1038/srep24458 (2016).

## Supplementary Material

Supplementary Information

## Figures and Tables

**Figure 1:**
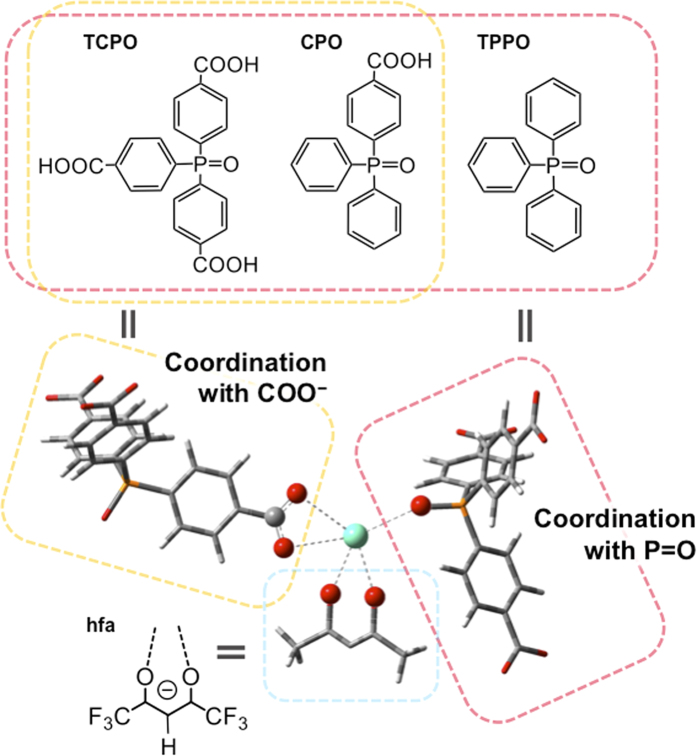
Structural images of Eu^III^ luminophore, Eu(hfa)_x_(TCPO)_y_, Eu(hfa)_x_(CPO)_y_ and Eu(hfa)_3_(TPPO)_2_ described using GaussView 5.0.

**Table 1:** Crystallographic data for Eu(hfa)_x_(TCPO)_y_.

Chemical formula	C_22.32_H_17.28_EuO_10.6_P
Formula weight	638.03
Crystal system	Triclinic
Space group	*P* -1(#2)
*a*/Å	10.7020(3)
*b*/Å	12.0041(3)
*c*/Å	14.5272(4)
*α*/deg	112.456(2)
*β*/deg	94.940(2)
*γ*/deg	101.867(2)
Volume/Å^3^	1659.69(8)
*Z*	2
*d*_calc_/g cm^−3^	1.277
Temperature/°C	−180
*μ* (Mo Kα)/ cm^−1^	19.747
max 2*θ*/deg	55.0
Reflections collected	30169
Independent reflections	7550
*R*_*1*_	0.0325
*wR*_2_	0.0847

[a] *R*_1_ = ∑ ||*F*_o_| ^−^ |*F*_c_||/∑ |*F*_o_|. [b] *wR*_2_ = [∑ w (*F*_o_^2^ –*F*_c_^2^)^2^/∑ *w* (*F*_o_^2^)^2^].

**Table 2:** Photophysical properties of each complexes in the solid state.

	Td( °C)	τ_obs_(ms)	τ_rad_ (ms)	τ_4f-4f_(%)	*k*_*r*_ (s^−1^)	*k*_*nr*_ (s^−1^)	Φπ_−_π* (%)	*η*(%)
Eu(hfa)_3_(TPPO)_2_	200	0.80	1.23	65	8.1 × 10^2^	4.4 × 10^2^	51	78
Eu(hfa)_3_(dpbp)	308	0.85	1.20	72	8.5 × 10^2^	3.2 × 10^2^	29	40
Eu(hfa)_x_(CPO)_y_	200	0.60	2.13	28	4.7 × 10^2^	1.2 × 10^3^	15	54
Eu(hfa)_x_(TCPO)_y_	450	0.61	1.77	34	5.7 × 10^2^	1.1 × 10^3^	20	59

Td is decomposition temperature. Φπ_−_π* is emission quantum yield excited at 355 nm. *η* is energy transfer efficiency from hfa moieties to Eu^III^ ions. Eu(hfa)_3_(TPPO)_2_: see ref. 39. Eu(hfa)_3_(dpbp): see ref. 40.

## References

[b1] Hou D. *et al.* Intense Blue Emission Phosphor BaCa_2_MgSi_2_O_8_: Eu^2+^ for Fluorescent Lamps. ECS J. Solid State Sci. Technol. 2, R79–R81 (2013).

[b2] Kuo T.-W., Huang C.-H. & Chen T.-M. Intense violet-blue-emitting Ba_2_AlB_4_O_9_Cl:Eu^2+^ phosphors for applications in fluorescent lamps and ultraviolet-light-emitting diodes. Appl. Opt. 49, 4202–4206 (2010). 20676174 10.1364/AO.49.004202

[b3] Huang C.-H., Kuo T.-W. & Chen T.-M. Thermally stable green Ba_3_Y(PO_4_)_3_:Ce^3+^,Yb^3+^ and red Ca_3_Y(AlO)_3_(BO_3_)_4_:Eu^3+^ phosphors for white-light fluorescent lamps. Opt. Express 19, 238–241 (2011). 10.1364/OE.19.0000A1 21263707

[b4] Wang J. *et al.* Solution-processible brilliantly luminescent Eu^III^ complexes with host-featured phosphine oxide ligands for monochromic red-light-emitting diodes. Chem. Eur. J. 20, 11137–11148 (2014). 25065610 10.1002/chem.201403244

[b5] Xu H. *et al.* A unique white electroluminescent one-dimensional europium(III) coordination polymer. J. Mater. Chem. C 3, 1893–1903 (2015).

[b6] Ahmed Z. & Iftikhar K. Efficient photoluminescent complexes of 400–1800 nm wavelength emitting lanthanides containing organic sensitizers for optoelectronic devices. RSC Adv. 4, 63696–63711 (2014).

[b7] Xu H., Zhu R., Zhao P. & Huang W. Monochromic Red-Emitting Nonconjugated Copolymers Containing Double-Carrier-Trapping Phosphine Oxide Eu^3+^ Segments: Toward Bright and Efficient Electroluminescence. J. Phys. Chem. C 115, 15627–15638 (2011).

[b8] Ling Q. *et al.* Non-Volatile Polymer Memory Device Based on a Novel Copolymer of N-Vinylcarbazole and Eu-Complexed Vinylbenzoate. Adv. Mater. 17, 455–459 (2005).

[b9] He H. *et al.* Controllable synthesis of Zn_2_GeO_4_ :Eu nanocrystals with multi-color emission for white light-emitting diodes. J. Mater. Chem. C 3, 5419–5429 (2015).

[b10] Rangari V. V. & Dhoble S. J. Synthesis and photoluminescence studies of Ba(Gd,Ln)B_9_O_16_:Eu^3+^ (Ln = La,Y) phosphors for n-UV LED lighting and display devices. J. Rare Earths 33, 140–147 (2015).

[b11] Du P., Krishna Bharat L. & Yu J. S. Strong red emission in Eu^3+^/Bi^3+^ ions codoped CaWO_4_ phosphors for white light-emitting diode and field-emission display applications. J. Alloys Compd. 633, 37–41 (2015).

[b12] Bellocchi G., Fabbri F., Miritello M., Iacona F. & Franzò G. Multicolor Depth-Resolved Cathodoluminescence from Eu-Doped SiOC Thin Films. ACS Appl. Mater. Interfaces 7, 18201–18205 (2015). 26258654 10.1021/acsami.5b05348

[b13] Kumar K. N., Vijayalakshmi L. & Ratnakaram Y. C. Energy transfer based photoluminescence properties of (Sm^3+^+Eu^3+^):PEO+PVP polymer films for Red luminescent display device applications. Opt. Mater. 45, 148–155 (2015).

[b14] Hasegawa Y. Photofunctional Lanthanoid Complexes, Coordination Polymers, and Nanocrystals for Future Photonic Applications. Bull. Chem. Soc. Jpn. 87, 1029–1057 (2014).

[b15] Kanazawa K., Nakamura K. & Kobayashi N. Electrochemical luminescence modulation in a Eu(III) complex-modified TiO_2_ electrode. J. Mater. Chem. C 3, 7135–7142 (2015).

[b16] Hirai Y. *et al.* Luminescent Coordination Glass: Remarkable Morphological Strategy for Assembled Eu(III) Complexes. Inorg. Chem. 54, 4364–4370 (2015). 25872587 10.1021/acs.inorgchem.5b00145

[b17] Hasegawa Y. *et al.* Enhanced Electric Dipole Transition in Lanthanide Complex with Organometallic Ruthenocene Units. J. Phys. Chem. A 119, 4825–4833 (2015). 25875051 10.1021/acs.jpca.5b01809

[b18] Daumann L. J. *et al.* New Insights into Structure and Luminescence of Eu (III) and Sm (III) Complexes of the 3,4,3-LI(1,2-HOPO) Ligand. J. Am. Chem. Soc. 137, 2816–2819 (2015). 25607882 10.1021/ja5116524 PMC4433002

[b19] Reddy M. L. P. & Sivakumar S. Lanthanide benzoates: A versatile building block for the construction of efficient light emitting materials. Dalton Trans. 42, 2663–2678 (2013). 23258556 10.1039/c2dt32470a

[b20] Ancel L., Gateau C., Lebrun C. & Delangle P. DNA Sensing by a Eu-Binding Peptide Containing a Proflavine Unit. Inorg. Chem. 52, 552–554 (2013). 23289864 10.1021/ic302456q

[b21] Debroye E. *et al.* Micellar self-assemblies of gadolinium(III)/europium(III) amphiphilic complexes as model contrast agents for bimodal imaging. Dalton Trans. 43, 3589–3600 (2014). 24402380 10.1039/c3dt52842a

[b22] da Silva F. F. *et al.* New lanthanide-CB[6] coordination compounds: relationships between the crystal structure and luminescent properties. Dalton Trans. 43, 5435–5442 (2014). 24522452 10.1039/c3dt52687a

[b23] Pacold J. I. *et al.* Direct Observation of 4f Intrashell Excitation in Luminescent Eu Complexes by Time-Resolved X-ray Absorption Near Edge Spectroscopy. J. Am. Chem. Soc. 136, 4186–4191 (2014). 24606064 10.1021/ja407924m

[b24] Biju S. *et al.* A Eu(III) Tetrakis(β-diketonate) Dimeric Complex: Photophysical Properties, Structural Elucidation by Sparkle/AM1 Calculations, and Doping into PMMA Films and Nanowires. Inorg. Chem. 53, 8407–8417 (2014). 25068684 10.1021/ic500966z

[b25] Caffrey D. F. & Gunnlaugsson T. Displacement assay detection by a dimeric lanthanide luminescent ternary Tb(III)–cyclen complex: high selectivity for phosphate and nitrate anions. Dalton Trans. 43, 17964–17970 (2014). 25374328 10.1039/c4dt02341b

[b26] Biju S., Eom Y. K., Bunzli J.-C. G. & Kim H. K. Biphenylene-bridged mesostructured organosilica as a novel hybrid host material for Ln^III^ (Ln = Eu, Gd, Tb, Er, Yb) ions in the presence of 2-thenoyltrifluoroacetone. J. Mater. Chem. C 1, 3454–3466 (2013).

[b27] Kitchen J. A. *et al.* Circularly Polarized Lanthanide Luminescence from Langmuir-Blodgett Films Formed from Optically Active and Amphiphilic Eu^III^-Based Self-Assembly Complexes. Angew. Chem. Int. Ed. 51, 704–708 (2012). 10.1002/anie.201106863 22162422

[b28] Neil E. R., Funk A. M., Yufit D. S. & Parker D. Synthesis, stereocontrol and structural studies of highly luminescent chiral tris-amidepyridyl-triazacyclononane lanthanide complexes. Dalton Trans. 43, 5490–5504 (2014). 24531186 10.1039/c3dt53000k

[b29] Shelton A. H., Sazanovich I. V., Weinstein J. a. & Ward M. D. Controllable three-component luminescence from a 1,8-naphthalimide/Eu(iii) complex: white light emission from a single molecule. Chem. Commun. 48, 2749–2751 (2012). 10.1039/c2cc17182a 22246467

[b30] Bünzli J.-C. G. On the design of highly luminescent lanthanide complexes. Coord. Chem. Rev. 293-294, 19–47 (2015).

[b31] Shavaleev N. M., Eliseeva S. V., Scopelliti R. & Bünzli J.-C. G. Tridentate Benzimidazole-Pyridine-Tetrazolates as Sensitizers of Europium Luminescence. Inorg. Chem. 53, 5171–5178 (2014). 24807159 10.1021/ic500267b

[b32] Lehr J., Beer P. D., Faulkner S. & Davis J. J. Exploiting lanthanide luminescence in supramolecular assemblies. Chem. Commun. 50, 5678–5687 (2014). 10.1039/c4cc01138d 24664123

[b33] Sykes D. *et al.* Sensitisation of Eu(III)- and Tb(III)-based luminescence by Ir(III) units in Ir/lanthanide dyads: evidence for parallel energy-transfer and electron-transfer based mechanisms. Dalton Trans. 43, 6414–6428 (2014). 24608523 10.1039/c4dt00292j

[b34] Sykes D. *et al.* d → f Energy Transfer in Ir(III)/Eu(III) Dyads: Use of a Naphthyl Spacer as a Spatial and Energetic ‘Stepping Stone’. Inorg. Chem. 52, 10500–10511 (2013). 24007190 10.1021/ic401410g PMC3971759

[b35] Eliseeva S. V. & Bünzli J.-C. G. Lanthanide luminescence for functional materials and bio-sciences. Chem. Soc. Rev. 39, 189–227 (2010). 20023849 10.1039/b905604c

[b36] Zhang H. *et al.* Highly luminescent and thermostable lanthanide-carboxylate framework materials with helical configurations. J. Mater. Chem. 22, 21210–21217 (2012).

[b37] Lee W. R. *et al.* Microporous Lanthanide-Organic Frameworks with Open Metal Sites: Unexpected Sorption Propensity and Multifunctional Properties. Inorg. Chem. 49, 4723–4725 (2010). 20450205 10.1021/ic902182x

[b38] Kataoka H. *et al.* Photo- and thermo-stable luminescent beads composed of Eu(III) complexes and PMMA for enhancement of silicon solar cell efficiency. J. Alloys Compd. 601, 293–297 (2014).

[b39] Hasegawa Y. *et al.* Absorption cross-section control of Eu(III) complexes for increase of amplified spontaneous emission excited by third harmonic of nanosecond Nd:YAG laser. J. Alloys Compd. 488, 578–581 (2009).

[b40] Miyata K. *et al.* Thermostable Organo-phosphor: Low-Vibrational Coordination Polymers That Exhibit Different Intermolecular Interactions. Chem Plus Chem 77, 277–280 (2012).

[b41] Friesen C. M., Montgomery C. D. & Temple S. A. J. U. The first fluorous biphase hydrogenation catalyst incorporating a perfluoropolyalkylether: [RhCl(PPh_2_(C_6_H_4_C(O)OCH_2_CF(CF_3_)(OCF_2_CF(CF_3_))_n_F))_3_] with *n *= 4–9. J. Fluor. Chem. 144, 24–32 (2012).

[b42] Václavík J. *et al.* AuI Catalysis on a Coordination Polymer: A Solid Porous Ligand with Free Phosphine Sites. Chem Cat Chem 5, 692–696 (2013).

[b43] Hasegawa Y. *et al.* Effect of Ligand Polarization on Asymmetric Structural Formation for Strongly Luminescent Lanthanide Complexes. Eur. J. Inorg. Chem. 2013, 5911–5918 (2013).

