# Supplementary material for: Hyper-stable organo-EuIII luminophore under high temperature for photo-industrial application
Source: Sci Rep. 2016 Apr 14;6:24458. doi: 10.1038/srep24458 (PMC4830997; doi:10.1038/srep24458)
Supplement: Supplementary Information [file srep24458-s1.pdf]

Supplementary Information

Hyper-stable organo-Eu^{III} luminophore under high temperature for photo-industrial application

Ayako Nakajima, Takayuki Nakanishi, Yuichi Kitagawa, Tomohiro Seki, Hajime Ito, Koji Fushimi and Yasuchika Hasegawa

Faculty of Engineering, Hokkaido University, N13 W8, Kita-ku, Sapporo,
Hokkaido 060-8626, Japan.

A. Nakajima et al, Fig. S1.

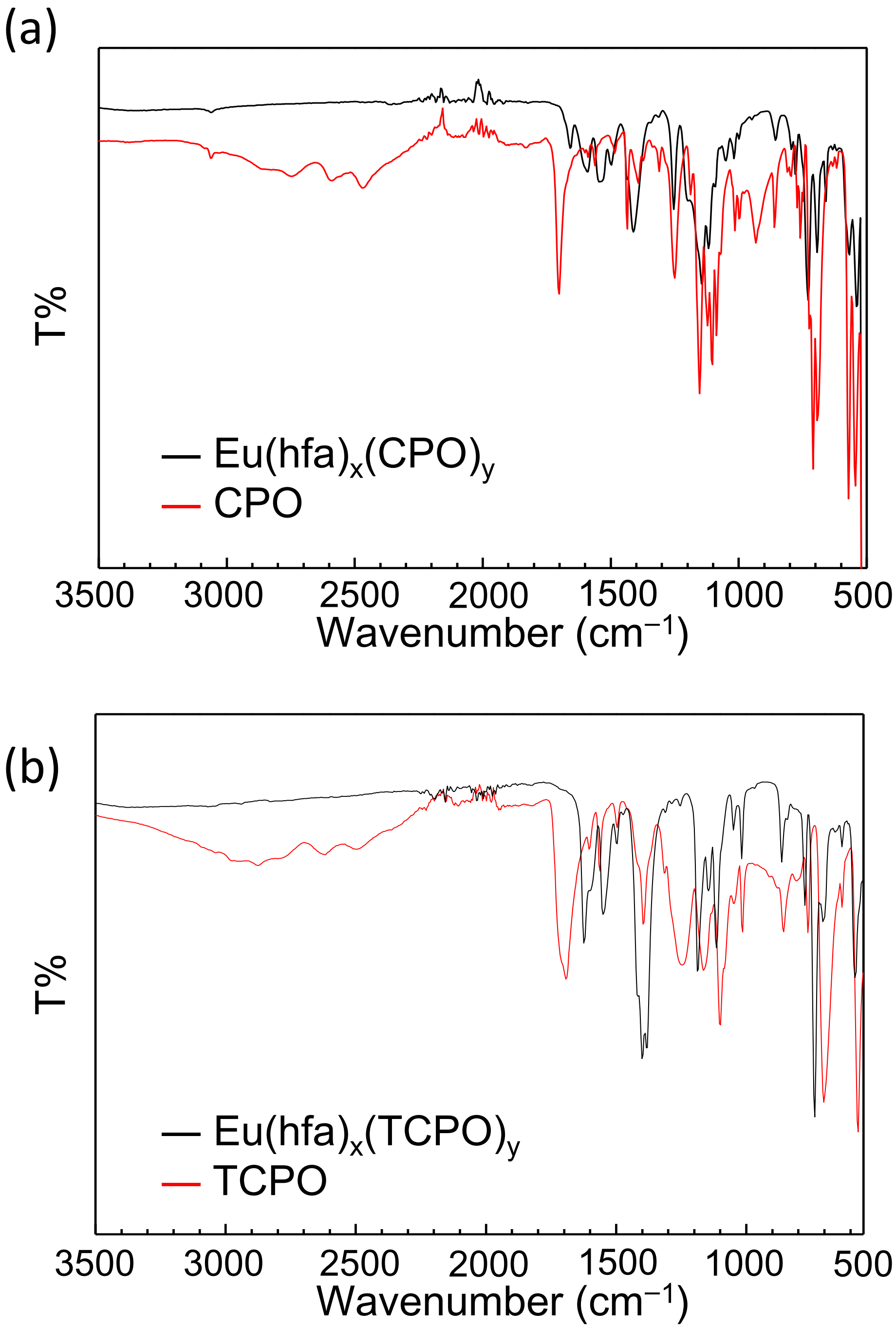

The IR spectra of (a) $Eu(hfa)_x(CPO)_y$ (black line), CPO (red line) and (b) $Eu(hfa)_x(TCPO)_y$ (black line), TCPO (red line).

A. Nakajima et al, Fig. S2.

(a)

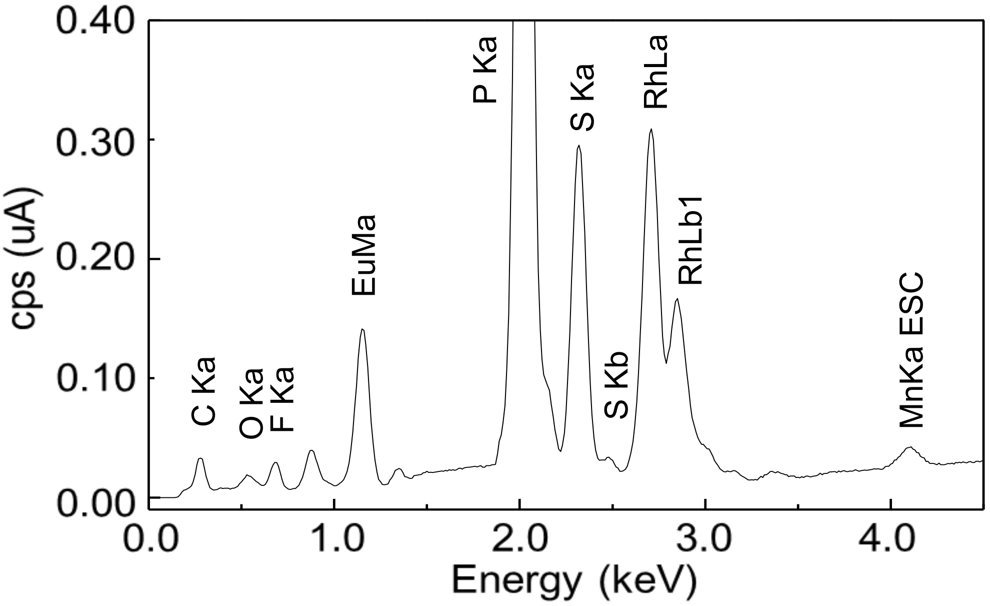

(b)

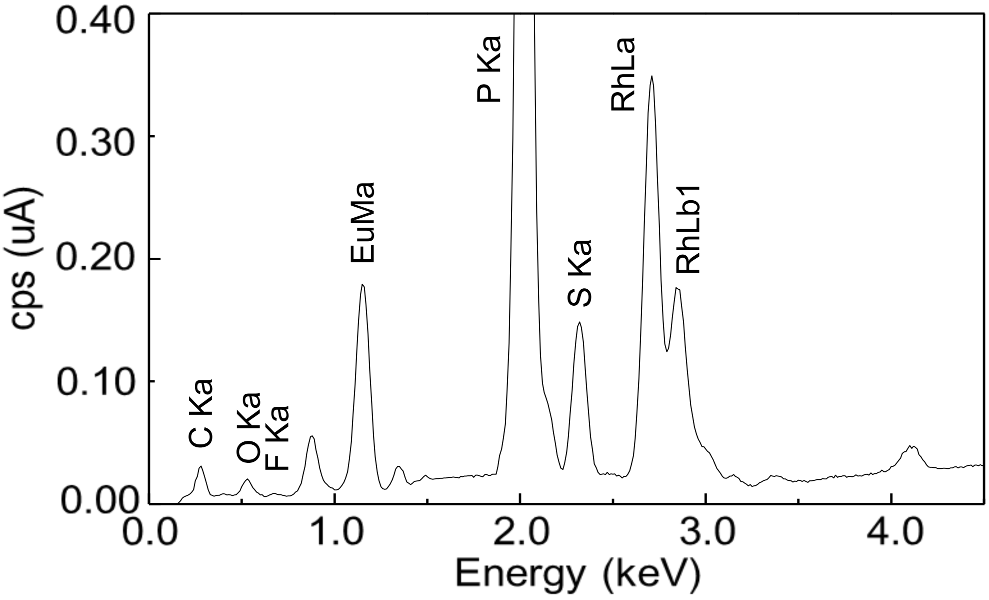

The EDX spectra of (a) $Eu(hfa)_x(CPO)_y$ and (b) $Eu(hfa)_x(TCPO)_y$.

A. Nakajima et al, Fig. S3.

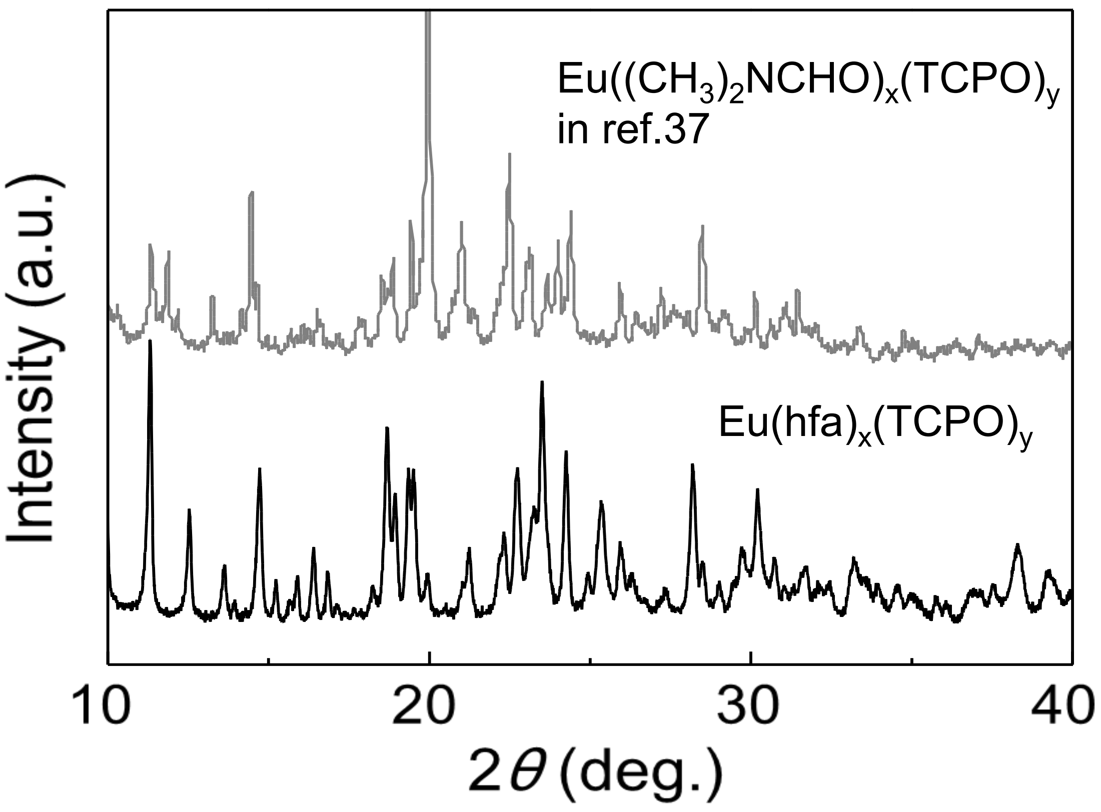

The XRD pattern of Eu(hfa)$_x$(TCPO)$_y$ (lower) compared with Eu((CH$_3$)$_2$NCHO)$_x$(TCPO)$_y$ (upper) synthesized according to ref. 37.

A. Nakajima et al, Fig. S4.

(a)

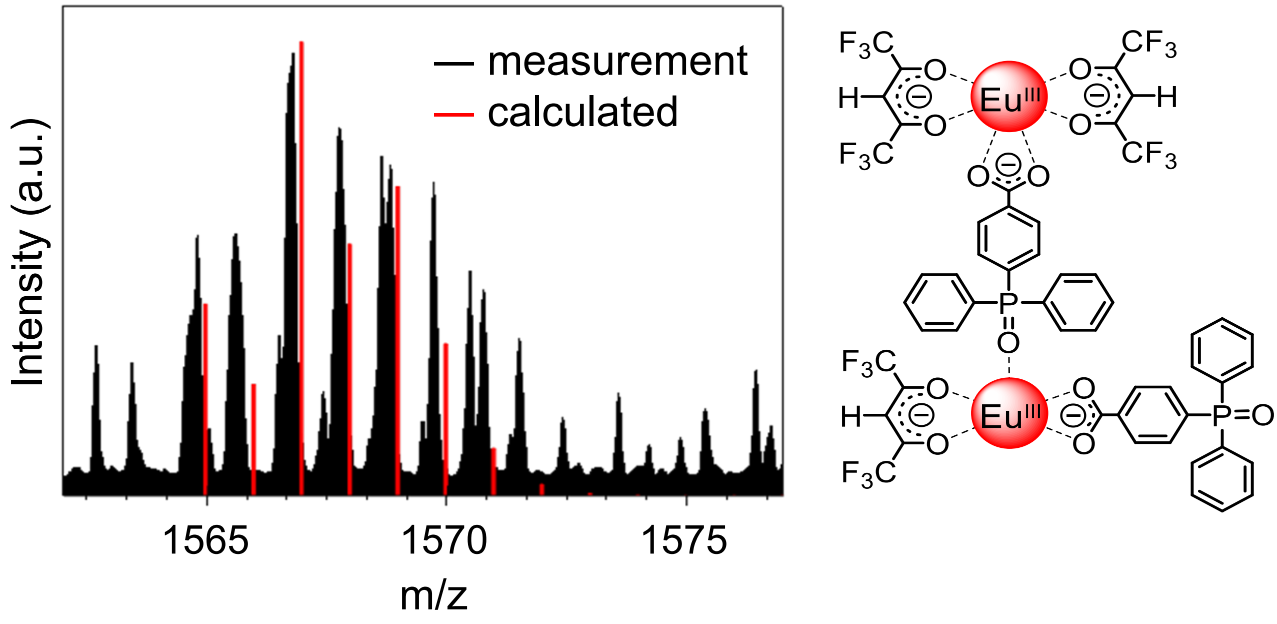

(b)

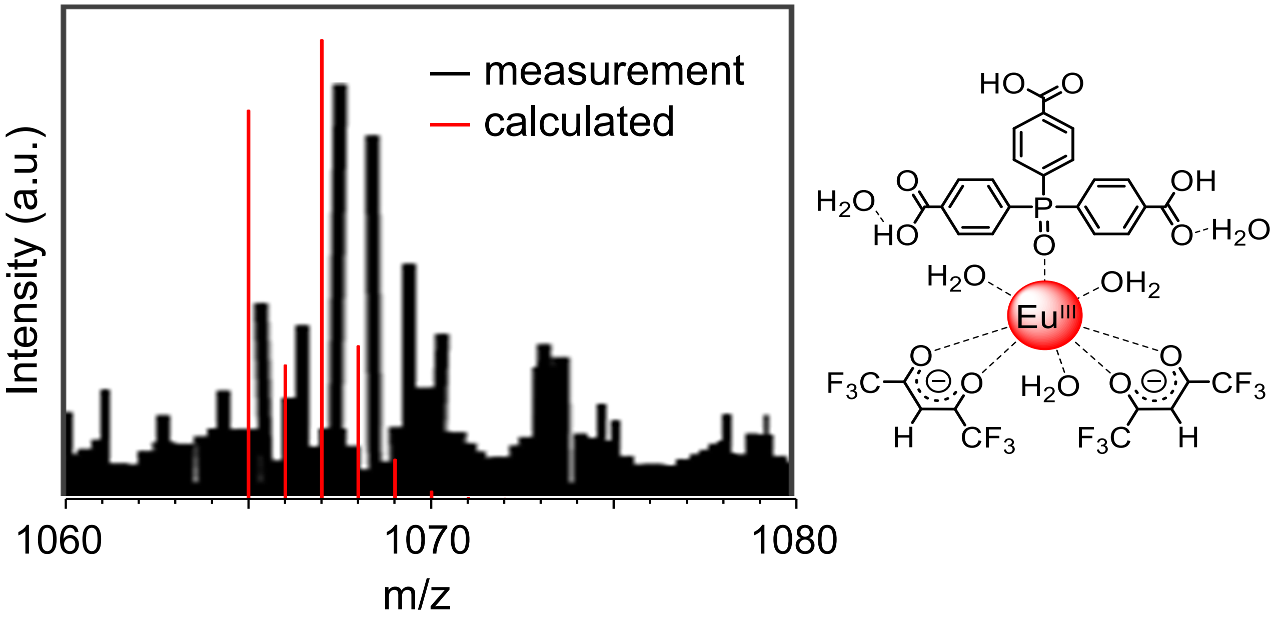

The fragment peaks of (a) Eu(hfa)$_x$(CPO)$_y$ and (b) Eu(hfa)$_x$(TCPO)$_y$ in FAB-MS and structural images.

A. Nakajima et al, Fig. S5.

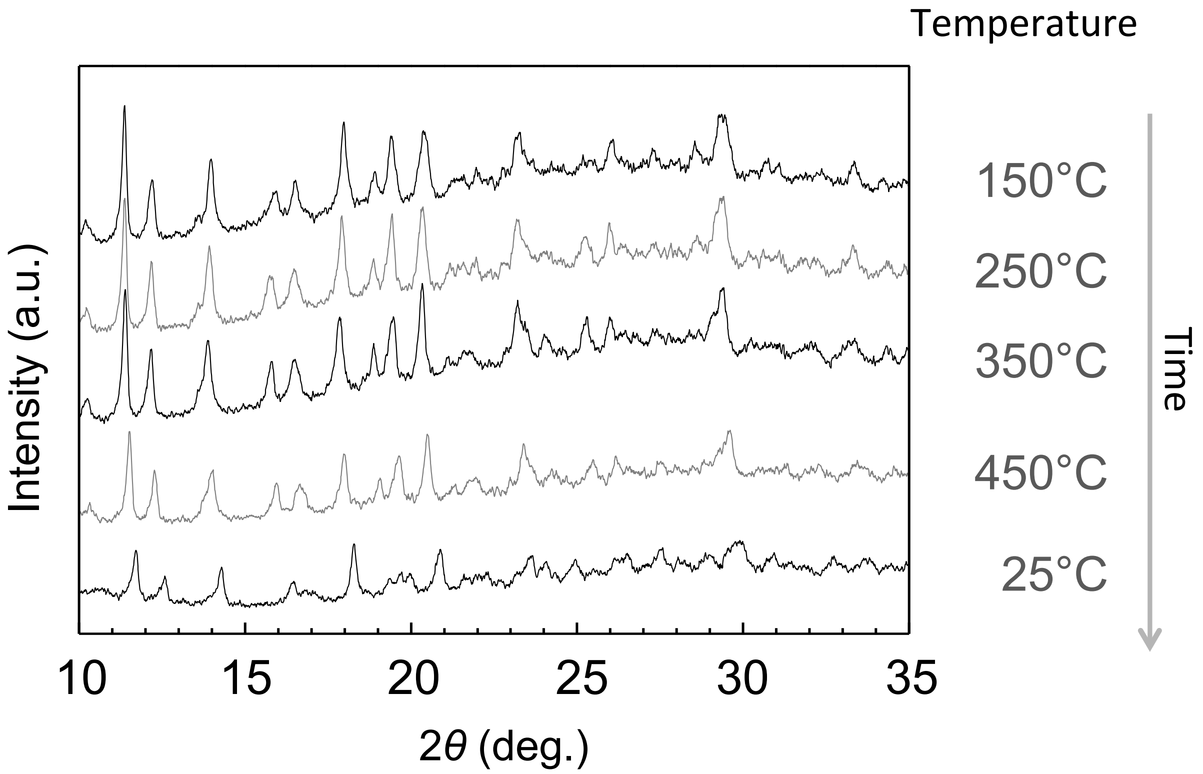

The diffraction peaks of the $Eu(hfa)_x(TCPO)_y$ in the XRD pattern at 150—450°C and at 25°C after heating up to 450°C.

A. Nakajima et al, Fig. S6.

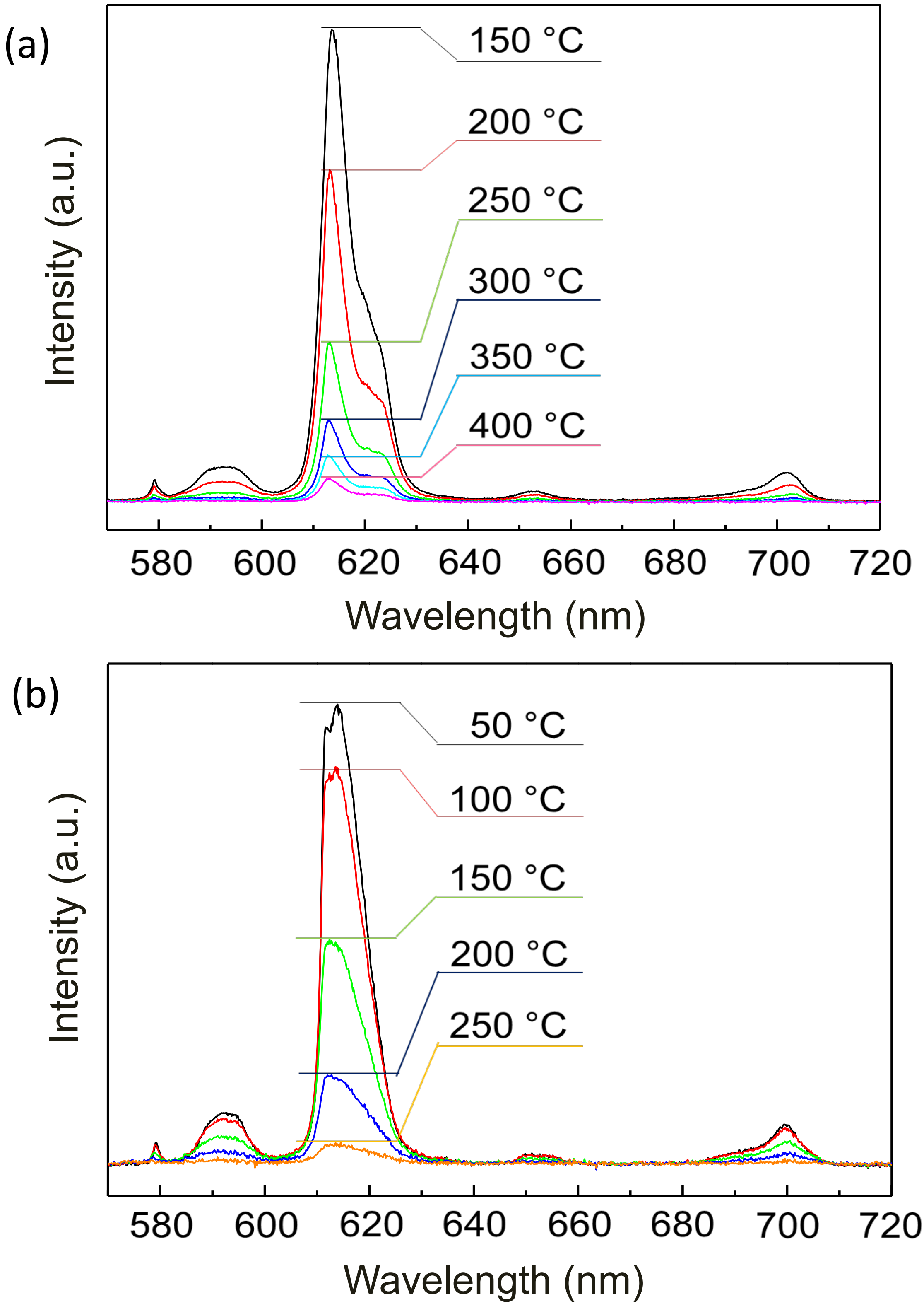

The emission spectra of (a) Eu(hfa)$_x$(TCPO)$_y$ and (b) Eu(hfa)$_x$(CPO)$_y$ measured by using Ocean Optics Inc. USB 4000 spectometer under control of temperature.

A. Nakajima et al, Fig. S7.

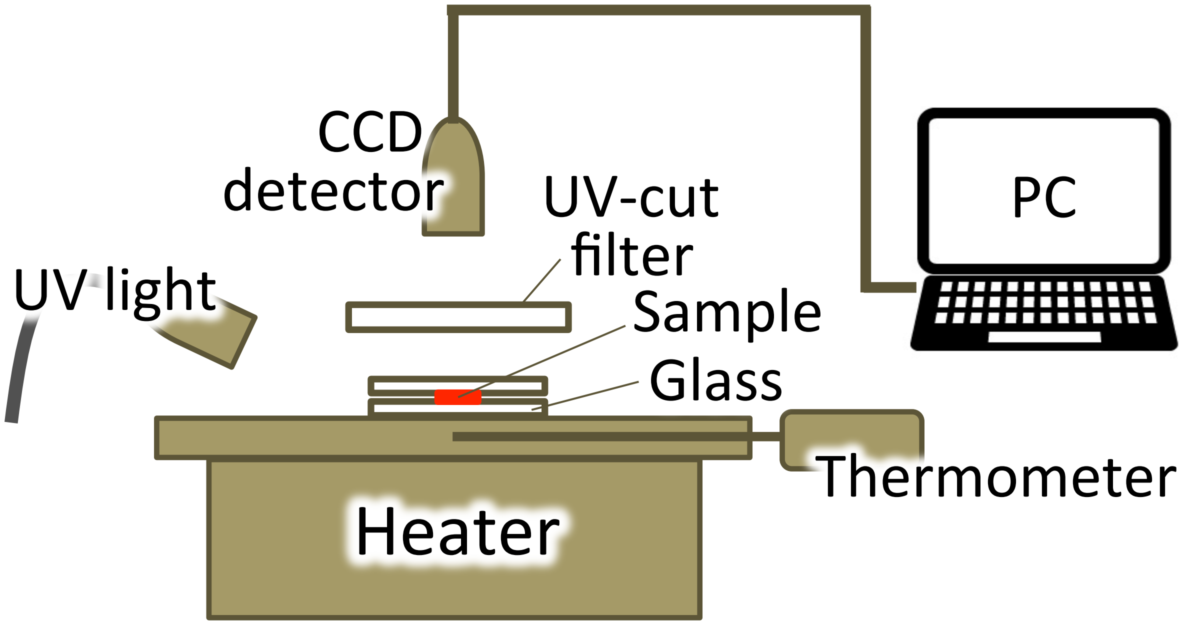

The image of set up as measuring temperature-independent emission spectra (Fig. S6).

A. Nakajima et al, Fig. S8.

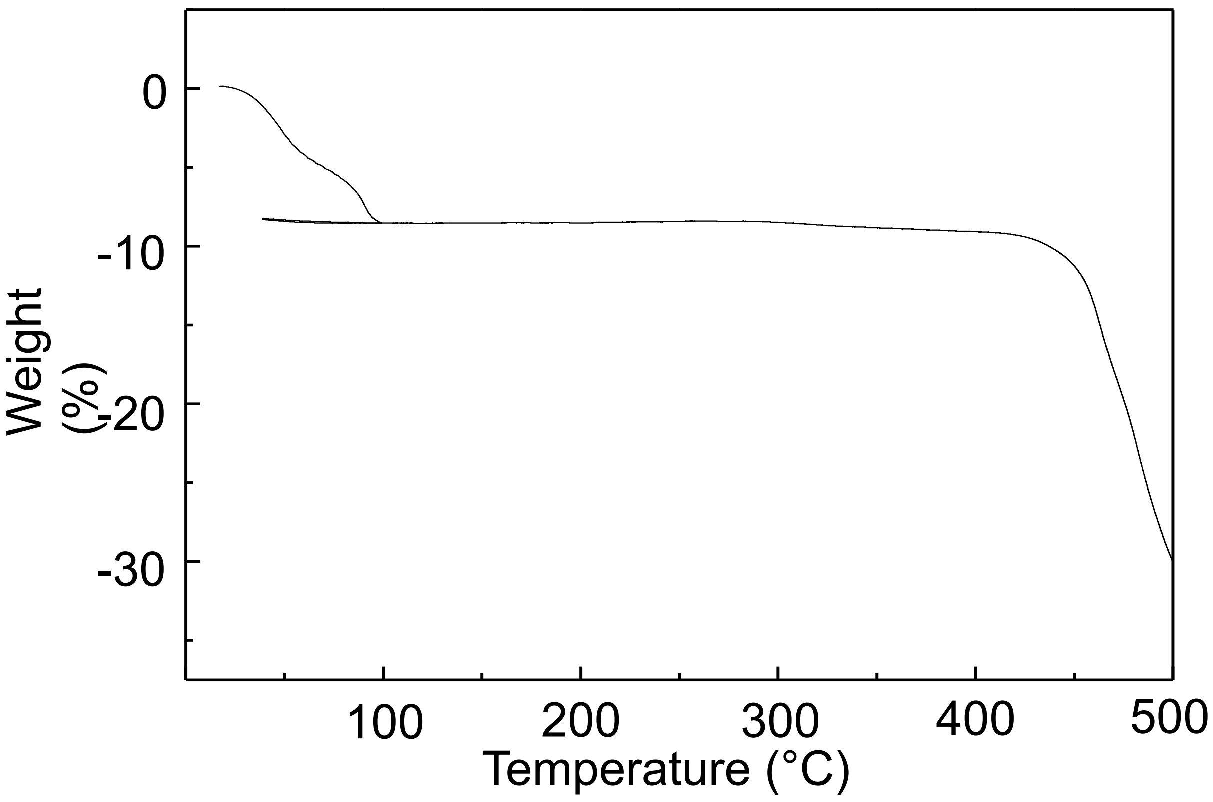

TGA profiles of $Eu(hfa)_x(TCPO)_y$ at first heating rate of 10°C min^{-1} up to 100°C, cooling rate of 10°C min^{-1} up to 40°C, and second heating rate of 1°C min^{-1} up to 500°C.

In order to remove the coordinated water, dehydration treatment of as-prepared $Eu(hfa)_x(TCPO)_y$ powder performed under vacuum (90°C, 2 h, under reduced pressure). The weight loss of as-prepared $Eu(hfa)_x(TCPO)_y$ in TG analysis (8% in Fig. S8) agrees with total weight of two water and two methanol molecules in $Eu(hfa)_x(TCPO)_y$ (10%).

A. Nakajima et al, Fig. S9.

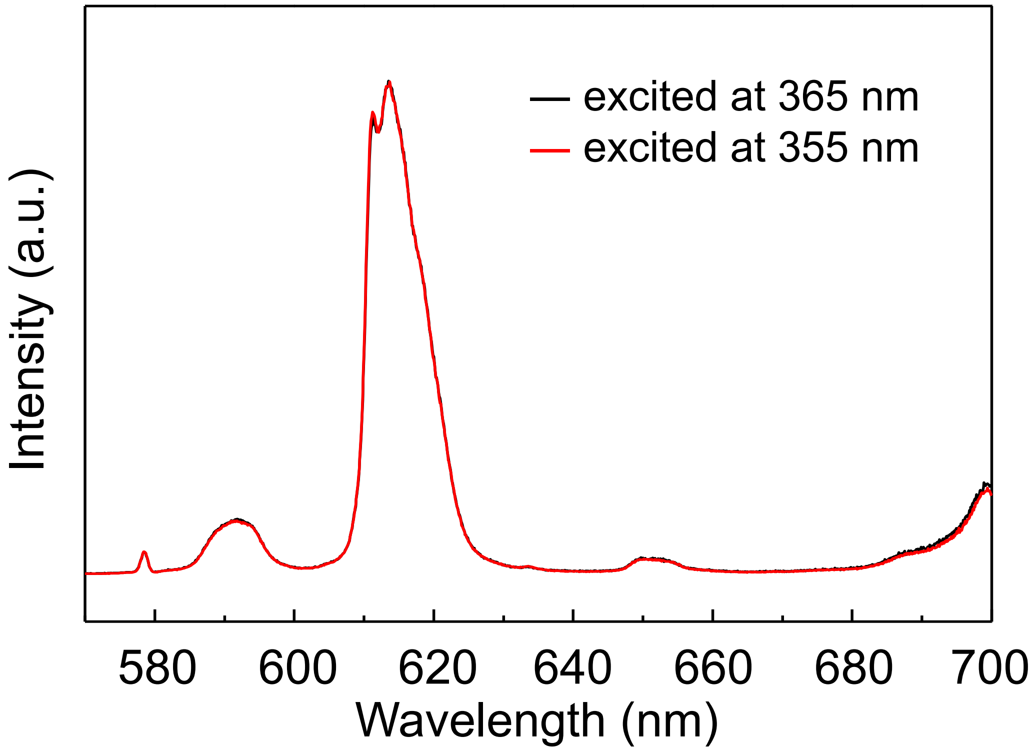

The emission spectra of Eu(hfa)$_x$(TCPO)$_y$ excited at 365 nm (black line) and 355 nm (red line) in the solid state.
